# Intraoperative rupture of an interlobar bronchial artery aneurysm: a case report

**DOI:** 10.1186/s13019-015-0313-y

**Published:** 2015-10-16

**Authors:** Yoshio Tsunezuka, Nobuyoshi Tanaka, Hideki Fujimori

**Affiliations:** Department of General Thoracic Surgery, Ishikawa Prefectural Central Hospital, Kuratsuki-higashi 2-1, Kanazawa, 920-8530 Japan

**Keywords:** Bronchial artery aneurysm, Rapture, Surgery, Percutaneous cardiopulmonary support

## Abstract

Here we report the rare case of an intraoperative bronchial artery aneurysm (BAA) rupture. An asymptomatic 52-year-old woman was found to have bilateral, multiple dilated bronchial arteries feeding the BAA that was further connected to the pulmonary artery on computed tomography and angiography. Transcatheter arterial embolization was thought not to be succeed. During a thoracoscopic procedure, the BAA ruptured suddenly and was treated with a thoracotomy under percutaneous cardiopulmonary support (PCPS). For anatomical complex BAA like the present case, the use of an open procedure and the preparation of PCPS are strongly recommended.

## Case presentation

It is recommended that bronchial artery aneurysm (BAA) be immediately treated to avoid risk of rupture. Generally, rupture will occur either before or after the surgical procedure and treatment aims to prevent or cease potentially fatal hemorrhage. Here, we present a rare case of a bronchial artery aneurysm (BAA) that ruptured intraoperatively.

**Fig. 1:**
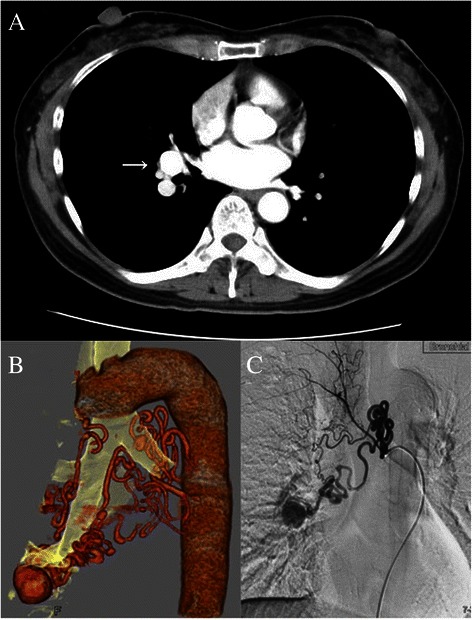
Preoperative CT scan showing the bronchial arterial aneurysm connected to the basal pulmonary artery (**a**), 3-D CT (**b**), and selective bronchial arteriography (**c**) revealing four tortuous feeding bronchial arteries and the saccular bronchial arterial aneurysm of the basal bronchus

A 52-year-old female patient, an ex-smoker, underwent a chest computed tomography (CT) scan before the surgical treatment of a right ovarian cyst and was found to have a mass shadow. Contrast-enhanced chest CT revealed a BAA of 17 mm in diameter, located between the middle and lower lobes of the lung and connected to the basal pulmonary artery, A8 (Fig. 1a). Angiography showed four bronchial arteries, bilateral and dilated, which were feeding the aneurysm (Fig. 1b, c). Transcatheter arterial embolization (TAE) was performed and we attempted to insert the smallest micro-coil catheter available (Excelsior™ SL-10 Microcatheter, Boston Scientific Co, USA) into the feeding bronchial arteries. However, the catheter advanced only slightly, approximately 6 cm and 2 cm into the left and right bronchial arteries respectively. It was therefore concluded that there were four interconnected dilated bronchial arteries feeding the BAA and that the aneurysm was in fact distant from the aorta. Thus, arterial embolization was considered too challenging and expensive, and an alternative surgical approach was indicated.

**Fig. 2:**
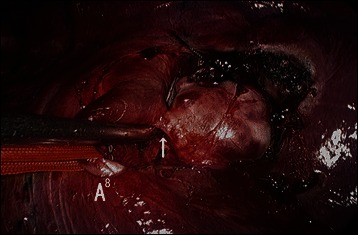
An intraoperative aspect view showing the bronchial arterial aneurysm connected to the pulmonary artery, (A^8^ , arrow)

**Fig. 3:**
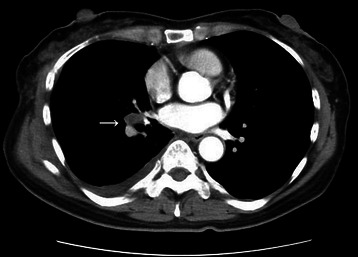
Postoperative CT scan showing the aneurysm decrease in size and thrombotic change

The patient initially underwent video-assisted bronchial artery ligation in the left lateral position. The bronchial arteries at the bifurcation of the trachea and below the azygous arch were ligated and incised. An inflamed lymph node was situated on the BAA, which was on the major fissure and was connected to the basal pulmonary artery A8 (Fig. 2). The lymph node on the BAA was resected and removed and the connecting artery was ligated with a 1-0 suture. During dissection of the connective tissue surrounding the BAA, the aneurysm ruptured and systolic blood pressure suddenly decreased to 60 mmHg. Therefore, a lateral thoracotomy was performed, compressing the BAA at the point of hemorrhage. The patient was placed in the hemi-lateral position , a 19 F and 17 F heparin-coated cannula (Bio-Medicus, Medtronic, Eden Prairie, MN, U.S.A) were inserted through the right femoral artery and vein. Under percutaneous cardiopulmonary (PCPS) support, the minor fissure – including the intermediate bronchus and central pulmonary artery – was partly closed using a large DeBakey pair of forceps. Our PCPS system consisted of a centrifugal pump (Mixflow pump, JMS, Japan), a membrane oxygenator (BIOCUBE-C 6000P, Nipro, Japan), and a reservoir. The aneurysm itself was sutured using continuous ‘over and over’ 4-0 non-absorbable polypropylene sutures. The total volume of blood loss was 1480 ml. The patient was discharged on the eighth postoperative day and there were no adverse events, recurrence of BAA, or complications at the 1-year follow-up. The CT scan showed that the aneurysm had decreased in size and undergone thrombotic change (Fig. 3).

## Discussion

BAA is a rare occurrence observed in less than 1 % of all cases of selective bronchial arteriography [1]. BAA development can be congenital (due to pulmonary sequestration or pulmonary agenesis) or acquired (due to lung disease, trauma, sepsis, inflammation, bronchiectasis, vasculitis, Behcet’s disease, or Osler–Weber–Rendu syndrome) [1]. Increased blood flow, high internal pressure, and degeneration and weakness of the vascular wall all play a contributing role towards the formation and progression of an aneurysm. The processes that lead to an aneurysmal rupture are unknown and diameter is not the sole incremental risk factor [2].

Surgical treatment comprises bronchial artery aneurysm excision, bronchial artery ligation, or lung resection [3]. BAA is classified on the basis of its location as one of two types: intrapulmonary or mediastinal. If an intrapulmonary BAA forms in the lung tissue, a pulmonary segmentectomy, lobectomy, or pneumonectomy can be performed. In particular, if a BAA exists concomitantly with bronchiectasis or pulmonary inflammation causing hemoptysis, a lung resection is the first surgical choice.

In general, a TAE is indicated as the first choice of treatment for many BAA cases as it is a less invasive procedure when gelatin sponges, detachable coils, occlusion balloons, or cyanoacrylate are employed [4]. A mediastinal BAA is particularly suitable for treatment with a TAE as the BAA is distal to the aorta and the feeding arteries are easily discernible. However, it is often challenging to perform a TAE if multiple feeding arteries form a complex network and the BAA is distant from the aorta. Moreover, a TAE should be performed to occlude not only the afferent artery but also the efferent artery, as collateral vessels may be a cause of revascularization [5]. Indeed, the present case is anatomically very rare with four responsible bronchial arteries interconnected to each other as well as to the pulmonary artery. As a result, it was endovascularly and surgically challenging, primarily due to the location of the BAA in an interlobar fissure distant from the aorta. Furthermore, the inflamed interlobar lymph node was obscured by the BAA. Due to the complex anatomy described above, a surgical procedure was indicated in this case. First, we ligated and incised the mediastinal bronchial arteries and the BAA remained taut, indicating additional arteries may have been responsible for feeding the aneurysm. The enclosed lymph node was then dissected from the aneurysm and the communicating vessel between the pulmonary artery and the aneurysm itself was successfully ligated. However, the internal pressure of the thin-walled aneurysm increased, resulting in rupture. Thus, it was necessary to clamp the central arteries flowing into the BAA to stop and prevent further hemorrhage. As it was not possible to clamp all the arteries, we chose to suture the aneurysmal wall. For aneurysms located in the left thorax, ligation of the bronchial arteries at the base is an effective treatment option. However, for aneurysms in the right thorax, collateral arteries should always be considered. Even if the BAA is not located near the aorta or does not rupture before surgery, an extracorporeal circulation system should be prepared in the event of an intraoperative BAA rupture.

## Conclusions

In a complex case such as that presented here, there is the potential for abrupt intraoperative BAA rupture. The use of an open procedure and the preparation of an extracorporeal circulation system are strongly recommended.

## Consent

Written informed consent was obtained from the patient for the publication of this report and any accompanying images.

## Abbreviations

BAA: Bronchial artery aneurysm
PCPS: Percutaneous cardiopulmonary support
CT: Computed tomography
